# Challenges in the diagnosis of chronic inflammatory demyelinating polyneuropathy

**DOI:** 10.1002/brb3.932

**Published:** 2018-02-07

**Authors:** Jeffrey A. Allen, Kenneth C. Gorson, Deborah Gelinas

**Affiliations:** ^1^ University of Minnesota Minneapolis MN USA; ^2^ Tufts University School of Medicine Boston MA USA; ^3^ Grifols Durham NC USA

**Keywords:** chronic inflammatory demyelinating polyneuropathy, clinical practice, diagnosis, guidelines, intravenous immunoglobulin

## Abstract

**Introduction:**

We explored adherence to the European Federation of Neurological Societies/Peripheral Nerve Society (EFNS/PNS) guidelines for the diagnosis and treatment of chronic inflammatory demyelinating polyneuropathy (CIDP) by reviewing data from a specialty pharmacy database.

**Materials and Methods:**

Clinical and electrophysiologic data were reviewed for 65 consecutive patients treated with intravenous immunoglobulin (IVIG) for CIDP. Three neuromuscular neurologists independently classified cases according to EFNS/PNS criteria as (1) fulfilling CIDP criteria; (2) non‐CIDP (neither clinical nor electrophysiologic criteria met); or (3) unknown (insufficient information).

**Results:**

Patients were treated by 31 different community neurologists in 14 states. Only seven patients (11%) met clinical and electrodiagnostic CIDP criteria. The remainder (89%) did not have CIDP (49%) or were unknown (40%). IVIG mean induction dose was 1.25 g/kg, mean maintenance dose 0.79 g/kg, and mean interval between infusions was 23 days.

**Conclusions:**

Adherence to EFNS/PNS CIDP diagnostic and treatment guidelines in the general neurologic community was poor. Improved education and awareness of widely available CIDP guidelines are recommended.

## 1 INTRODUCTION

Chronic inflammatory demyelinating polyneuropathy (CIDP) is an immune‐mediated neuropathy with a reported prevalence as high as 8.9 per 100,000 (Laughlin et al., 2009; Lunn, Manji, Choudhary, Hughes, & Thomas, 1999). The diagnosis of CIDP is based on clinical, electrophysiologic, and supportive or exclusionary data. However, misdiagnosis has been reported to occur in almost half of cases (Allen & Lewis, 2015; Cornblath, Gorson, Hughes, & Merkies, 2013). CIDP diagnostic and treatment guidelines can improve diagnostic accuracy but may be underutilized during routine clinical care.

The European Federation of Neurological Societies/Peripheral Nerve Society (EFNS/PNS) guidelines for the diagnosis of CIDP (Van den Bergh et al., 2010) are commonly accepted consensus‐derived criteria that capture both typical and atypical clinical variants of CIDP (Rajabally, Fowle, & Van den Bergh, 2015). In this study, we reviewed IVIG utilization data from a national specialty pharmacy database to evaluate diagnostic accuracy in the community as defined by EFNS/PNS guidelines and to understand if treatment was evidence‐based.

## 2 MATERIALS AND METHODS

### 2.1 Sampling

This was a retrospective data review of consecutive community patients newly diagnosed with CIDP who received IVIG treatment for the first time and were referred to aspecialty pharmacy for home infusion of IVIG. The review included clinical and electrodiagnostic data as well as cerebrospinal fluid, MRI, nerve biopsy, and prescribed treatment data when available. Copernicus Group IRB (Research Triangle Park, NC) reviewed this study and determined that the study met the criteria for an IRB exemption under the United States Code of Federal Regulations Title 45 Part 46 and a waiver of the informed consent requirement. All patients signed a waiver of consent.

### 2.2 Data collection and review

Pertinent positive and negative data, including IVIG dosing information, were compiled and anonymized by one reviewer (JAA) and recorded without interpretation into a CareLogic^®^ database (Qualifacts Systems Inc., Nashville, TN, USA).

Three reviewers used EFNS/PNS guidelines to independently evaluate each case. “Typical” and “atypical” variants were included. Typical CIDP was defined as chronically progressive, stepwise, or recurrent symmetric proximal and distal weakness and sensory dysfunction of all extremities, developing over at least 2 months, with possible cranial nerve involvement and diminished or absent deep tendon reflexes in all extremities (Van den Bergh et al., 2010). Atypical CIDP was defined according to EFNS/PNS criteria and included patients with predominantly distal, asymmetric, pure motor, or pure sensory variants (Van den Bergh et al., 2010). Electrodiagnostic EFNS/PNS criteria for CIDP (Van den Bergh et al., 2010) were applied to the review of the nerve conduction data. Final diagnosis was achieved by consensus among reviewers. Patients were classified as “CIDP” if all reviewers agreed that clinical and electrodiagnostic EFNS/PNS criteria were met (definite, probable, or possible). Patients fulfilling even minimal CIDP diagnostic criteria (possible) were categorized as CIDP. Similarly, all reviewers agreed when patients were stratified into a non‐CIDP category. If the available data were inadequate to reach consensus, cases were classified as “unknown.”

## 3 RESULTS

### 3.1 Sampling

Sixty‐five consecutive patients were diagnosed with CIDP and treated with IVIG by 31 neurologists in 14 states. The mean age was 60 years, and 55% were men. In general, patients were evenly distributed across study sites (range: 1–4). However, 19 neurologists referred one patient, and one neurologist contributed 19 patients.

### 3.2 Electrodiagnostic and clinical evaluation by reviewer

Interpretation of clinical and electrophysiologic data by reviewer is shown in Table 1. Although there was some variability in clinical classification among reviewers, interpretation of electrophysiologic criteria was more consistent. Final diagnostic classification took into consideration available supportive data, but the diagnosis of CIDP required that clinical and electrophysiologic criteria were met. Cerebrospinal fluid was sampled in only 13 patients (seven with cyto‐albuminologic dissociation), lumbosacral MRI in 15 patients (four with nerve root enlargement or enhancement), and nerve biopsy in three (two with histopathologic evidence of a demyelinating polyneuropathy). Preconsensus categorization of CIDP varied between 8% and 14% and non‐CIDP between 45% and 60%. Differences among reviewers reflected varying levels of confidence of assignment to the unknown category when definitive clinical data were not available. Consensus ratings arrived at unanimous agreement of CIDP classification (11%). Minimal discordancy remained in the non‐CIDP and unknown categories, categorized according to agreement on two of three ratings.

**Table 1:** Independent reviewer assessments^a^ (*N*  =  65)

	Reviewer		
	1 *n* (%)	2 *n* (%)	3 *n* (%)
Clinical criteria			
Typical CIDP	8 (12)	3 (5)	7 (11)
Atypical CIDP	22 (34)	9 (14)	20 (31)
Not consistent with CIDP phenotype	35 (54)	53 (82)	38 (58)
Electrophysiologic criteria			
Definite CIDP	11 (17)	12 (18)	12 (18)
Probable CIDP	1 (2)	3 (5)	0 (0)
Possible CIDP	8 (12)	7 (11)	13 (20)
Not consistent with CIDP	45 (69)	43 (66)	40 (62)
Exclusionary diagnosis present^b^	7 (11)	7 (11)	7 (11)
Final diagnosis of CIDP	7 (11)	5 (8)	9 (14)
Final diagnosis of non‐CIDP	34 (52)	39 (60)	29 (45)
Final diagnosis of Unknown	24 (37)	21 (32)	27 (42)
Final consensus diagnosis of CIDP	7 (11)	7 (11)	7 (11)
Final consensus diagnosis of non‐CIDP	33 (52)	37 (57)	36 (55)
Final consensus diagnosis of Unknown	25 (37)	21 (32)	22 (34)

CIDP, Chronic inflammatory demyelinating polyneuropathy.

a Sums may not equal 100% due to rounding.

b Includes anti‐MAG neuropathy (*n*  =  3), multifocal motor neuropathy (*n*  =  1), Lyme (*n*  =  1), B12 deficiency (*n*  =  1), and diabetic lumbosacral radiculoplexus neuropathy (*n*  =  1) (not mutually exclusive).

### 3.3 EFNS/PNS diagnostic categories

**Figure 1:**
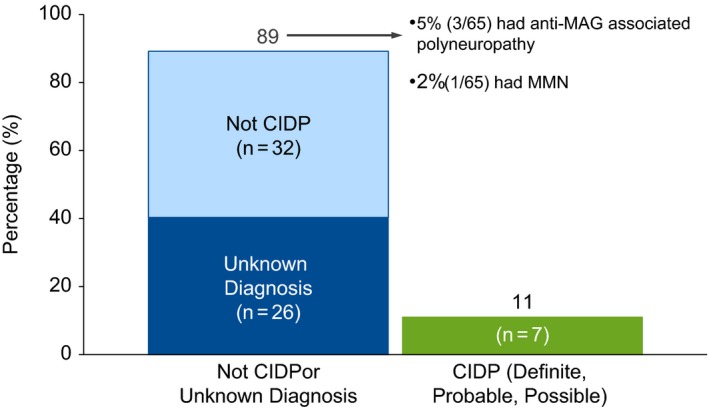
Diagnostic prevalence of CIDP. CIDP, chronic inflammatory demyelinating polyneuropathy; MAG, myelin‐associated glycoprotein; MMN, multifocal motor neuropathy

Only 11% (7/65) of cases had sufficient documentation to confirm a diagnosis of CIDP (Figure 1). The majority of cases did not have CIDP (49%, 32/65) or were unable to be classified due to incomplete data and therefore labeled unknown (40%, 26/65). Three of the 32 non‐CIDP patients were reclassified as antimyelin‐associated glycoprotein (MAG) neuropathy and one as multifocal motor neuropathy (MMN). The unknown patients were classified as such due to incomplete clinical or electrophysiologic data. From the single site that contributed 19 cases, none had CIDP. When these cases were excluded, the percentage of patients with confirmed CIDP increased to 15% (7/46), while 37% (17/46) did not have CIDP, and 48% (22/46) were classified as unknown.

### 3.4 Dosing

Intravenous immunoglobulin dosing data are presented in Table 2. The mean interval between the induction and the first maintenance dose was 25 days (range 2–59), and the interval between all infusions was 23 days (range 12–43). When compared with IVIG in CIDP Efficacy (ICE) protocol dosing, 70% of patients received induction doses <2 gm/kg, and 61% received maintenance infusions <1 gm/kg every 3 weeks. No dosing differences were seen in patients with confirmed CIDP, non‐CIDP, or unknown diagnosis (data not shown).

**Table 2:** Dosing (*N*  =  64^a^)

IVIG Dose Type	Mean (range) g/kg	Relationship to ICE Dosing^b^,^c^ *n* (%)		
		Below	Above	Equivalent
Induction	1.25 (0.3–2.5)	45 (70)	16 (25)	2 (3)
Maintenance	0.79 (0.2–2.4)	39 (61)	19 (30)	6 (9)

IVIG, intravenous immunoglobulin; ICE, Immune Globulin Intravenous CIDP Efficacy.

a Dosing data for one patient were discarded because infusion dates were illegible.

b Induction dose  =  2 g/kg.

c Maintenance dose  =  1 g/kg every 3 weeks or equivalent.

## 4 DISCUSSION

This study demonstrates that adherence to CIDP diagnostic guidelines within the community is poor. Only 11% of patients satisfied minimum EFNS/PNS diagnostic criteria. The true frequency of CIDP in our real‐world patient sample may be >11%, as more than one‐third of the available data were incomplete, or relevant abnormalities were poorly documented. While this observation limits the certainty with which we can understand the rate of CIDP misdiagnosis in the community, it nonetheless raises troubling concerns. In these cases, the most basic examination findings (e.g., degree and distribution of weakness, pattern and modality of sensory loss, and deep tendon reflexes findings) often were not recorded, electrophysiologic studies were inadequately performed or interpreted, and supportive diagnostic studies were not carried out, yet these patients were diagnosed and treated as CIDP. We also observed that based on the available data there was high diagnostic certainty that at least 49% did not have CIDP. These observations are disconcerting. We appreciate that treatment trials may have been reasonable in some with equivocal diagnostic findings or when a clear distinction between other immune‐mediated neuropathies cannot be made, as in the case of the patient reclassified as MMN. However, the majority of misdiagnosed patients had no such uncertainty. Approximately half of the patients had clinical, electrophysiologic, or exclusionary data that unequivocally should have precluded the diagnosis of CIDP.

We acknowledge that inclusion of 19 patients from one provider, none of whom had CIDP, influenced the percentage of patients in each diagnostic category. When these patients were excluded, the frequency of confirmed CIDP increased to only 15%, and 37% did not have CIDP. We elected to include the 19 misdiagnosed patients from the single provider into our analysis for several reasons. First, our intention was to report a real‐world sample of consecutive patients from a specialty pharmacy for home infusion of IVIG. Second, it raises concerns that while the overall frequency of CIDP misdiagnosis is high, there may be regional pockets and individual practitioners within the United States that are more prone to overdiagnosis. This outlier may call attention to a systemic issue that is not yet fully appreciated.

The frequency of CIDP misdiagnosis previously has been reported to be as high as 47% (Allen & Lewis, 2015). All patients in that study were evaluated at a tertiary referral center for another opinion. Conversely, patients in the present study were diagnosed and treated for presumed CIDP within the community prior to diagnostic reevaluation. The frequency of misdiagnosis highlights diagnostic challenges inherent to CIDP and emphasizes the benefit of obtaining an early expert opinion, especially for cases that are “atypical” or with equivocal diagnostic findings.

We acknowledge the possibility that physicians may have used the CIDP diagnostic billing code to secure IVIG approval for the treatment of non‐reimbursable conditions. Until recently, there was no ICD‐9 or ICD‐10 code for MMN or anti‐MAG inflammatory neuropathy. It is possible that the treating physicians knew they were not treating CIDP but were unable to classify correctly and desired to administer a trial of IVIG. If the four patients in this study (three with MAG inflammatory neuropathy and one MMN) are thus included as “CIDP definite, probable, or possible,” then the percentage of confirmed cases increases from 11% (7/65) to 17% (11/65). We consider this an unlikely explanation for our findings because in all referring clinical notes the codes matched the documented clinical conclusion.

All patients in this series were treated for presumptive CIDP, yet 70% of IVIG loading doses and 61% of maintenance regimens were below evidence‐based trial data (Hughes et al., 2008). There are no IVIG dosing studies, but the ICE trial showed unequivocal benefit when IVIG was administered at a loading dose of 2 gm/kg followed by 1 gm/kg every 3 weeks (Hughes et al., 2008). While justification for dosing above the ICE regimen might be understandable in patients who failed to improve with standard dosing, initial underdosing should be avoided as it may lead to an incorrect presumption of treatment failure. Improved adherence to widely available guidelines may minimize these avoidable errors.

Our study has several limitations. The EFNS/PNS criteria may not capture all patients with CIDP. We attempted to minimize author misclassification by including both “typical” and “atypical” clinical variants and of “possible” electrodiagnostic criteria. Although EFNS/PNS sensitivity for “definite” CIDP is 73% (specificity 88%), a “possible” diagnosis improves sensitivity to 91%, albeit at the cost of specificity (66%) (Breiner & Brannagan, 2014). Our liberal inclusion of atypical CIDP variants may be one reason for the variability in clinical classification among our reviewers. EFNS/PNS clinical criteria are intentionally broad such that all variants are captured under the CIDP umbrella. While this gives clinicians the autonomy to diagnosis CIDP with less restriction, descriptive criteria are inherently vulnerable to errors of assimilation. The required subjectivity of the clinical criteria highlights the importance of interpreting the clinical features within the context of all the available data, including the electrophysiologic findings and supportive data. Our observation that there is minimal evaluator disagreement of electrophysiologic data comes as no surprises, as the rigid nature of electrodiagnsotic criteria is less vulnerability to interpretive bias provided the predefined rules are followed. The observation that our final diagnostic conclusions also had minimal variability reinforces the notion that as a community there is a need to improve the way “atypical” symptoms and signs are reconciled with electrophysiologic data. These should be interpreted together, with only the sum leading to a final diagnosis of CIDP. We recommend future CIDP guidelines take these observations into account when constructing diagnostic criteria.

We appreciate that because our data were collected from a single specialty pharmacy, it may not be representative of the overall level of care of CIDP in all parts of the United States or by all clinicians that take care of patients with CIDP. With this caveat in mind, our findings do provide useful insight into the diagnostic and treatment practices in one real‐world setting. The extent to which these findings can be systemically extrapolated requires further study.

## 5 CONCLUSIONS

Chronic inflammatory demyelinating polyneuropathy is commonly misdiagnosed and insufficiently treated with IVIG in general community practice, at least according to the documentation used to obtain approval for IVIG treatment. Widely accepted and readily available CIDP diagnostic and treatment guidelines are underutilized during routine clinical care, highlighting the need for improved education and awareness of existing guidelines. Review of the records showed poor documentation and interpretation of bedside neuromuscular and electrodiagnostic findings. This study raises concerns about the adequacy of neurological training required for appropriate management of neuromuscular diseases. We urge greater adherence to published diagnostic criteria and proper use of proven treatment protocols when caring for patients with CIDP.

## CONFLICT OF INTEREST

None declared.
